# A SUMO-interacting motif activates budding yeast ubiquitin ligase Rad18 towards SUMO-modified PCNA

**DOI:** 10.1093/nar/gks892

**Published:** 2012-10-02

**Authors:** Joanne L. Parker, Helle D. Ulrich

**Affiliations:** Clare Hall Laboratories, Cancer Research UK London Research Institute, Blanche Lane, South Mimms, Hertfordshire EN6 3LD, UK

## Abstract

SUMO-targeted ubiquitin ligases (STUbLs) recognize sumoylated proteins as substrates for ubiquitylation and have been implicated in several aspects of DNA repair and the damage response. However, few physiological STUbL substrates have been identified, and the relative importance of SUMO binding versus direct interactions with the substrate remains a matter of debate. We now present evidence that the ubiquitin ligase Rad18 from *Saccharomyces cerevisiae*, which monoubiquitylates the sliding clamp protein proliferating cell nuclear antigen (PCNA) in response to DNA damage, exhibits the hallmarks of a STUbL. Although not completely dependent on sumoylation, Rad18’s activity towards PCNA is strongly enhanced by the presence of SUMO on the clamp. The stimulation is brought about by a SUMO-interacting motif in Rad18, which also mediates sumoylation of Rad18 itself. Our results imply that sumoylated PCNA is the physiological ubiquitylation target of budding yeast Rad18 and suggest a new mechanism by which the transition from S phase-associated sumoylation to damage-induced ubiquitylation of PCNA is accomplished.

## INTRODUCTION

Ubiquitin and the small ubiquitin-related modifier (SUMO) are small post-translational protein modifiers that alter the properties of their targets by affecting their activities, interactions, stabilities or intracellular localization (1). Despite non-overlapping conjugation machineries, there is extensive cross-talk between the two modification systems (2). A growing number of proteins are being identified as targets of both ubiquitin and SUMO. One example is IκBα, an inhibitor of the transcriptional activator NF-κB. IκBα is either ubiquitylated or sumoylated on the same lysine, but with opposite consequences for protein stability (3). A rather different relationship applies to a class of ubiquitin ligases (E3s) that recognize sumoylated targets as substrates for ubiquitylation and have been implicated in various aspects of DNA repair and genome maintenance (4–9). These SUMO-targeted ubiquitin ligases (STUbLs) are RING finger E3s harbouring conserved SUMO interaction motifs (SIMs), which consist of a hydrophobic core flanked by several acidic residues (10). By interacting predominantly with poly-SUMO chains, they mediate ubiquitylation of the SUMO moieties themselves, as well as the proteins to which these are attached. Hence, sumoylation can serve as a signal for subsequent ubiquitylation, often followed by proteasome-mediated degradation. Despite a profound influence on the homeostasis of SUMO conjugates in the cell, few physiological STUbL substrates have been identified to date.

Post-translational modifications of the budding yeast sliding clamp protein, proliferating cell nuclear antigen (PCNA), present a unique example of how ubiquitin and SUMO cooperate in the context of DNA replication and repair (11). In response to replication-stalling DNA damage, PCNA is monoubiquitylated at a highly conserved lysine, K164, by the E2–E3 complex Rad6–Rad18 (12). This promotes the recruitment of a class of specialized polymerases capable of using damaged DNA as a template for translesion synthesis (13–15). Extension to a polyubiquitin chain activates an error-free pathway of damage bypass that likely involves template switching (12). In contrast to ubiquitylation of PCNA, which is common to all eukaryotes, modification by SUMO appears to be less prevalent. In *Saccharomyces cerevisiae*, the SUMO E3 Siz1 promotes attachment of the SUMO homologue Smt3 mainly to K164 (12). K127 is modified to a lesser degree in a Siz1-independent manner (16). Sumoylation of budding yeast PCNA during S phase prevents unscheduled recombination events by enhancing the binding of an anti-recombinogenic helicase, Srs2 (17,18). Hence, the modification enables ubiquitin-dependent damage bypass by blocking alternative processing pathways.

The cooperation between SUMO and ubiquitin in orchestrating lesion bypass raises the question of how the transition from the S phase-associated sumoylated form of PCNA to the damage-induced ubiquitylated form is accomplished. The ubiquitin E3 Rad18, which is rate limiting for both mono- and polyubiquitylation of PCNA (19,20), is likely to play a critical role in this process. Intriguingly, a previous report suggested that Rad18 physically interacts with the SUMO E2 Ubc9 (12). We have now identified a SIM in Rad18 that strongly stimulates its ubiquitin ligase activity towards the sumoylated form of PCNA. We propose that budding yeast Rad18 is adapted to act primarily on sumoylated PCNA and discuss the implications for the switch between the two modifications in response to DNA damage.

## MATERIALS AND METHODS

### Yeast strains

All experiments involved the use of isogenic strains. *^His^POL30, siz1Δ*, *rad18Δ* and the lysine mutants of *POL30* have been described previously (15,19). The 9myc epitopes were appended by a polymerase chain reaction strategy to *RAD18 wildtype (WT)* or SIM* alleles that had been inserted into the *URA3* locus of *rad18Δ* on integrative vectors derived from YIplac211, bearing the *RAD18* promoter.

### Proteins

Recombinant budding yeast ^His^PCNA, ^His^Ub-PCNA, Replication Factor C (RFC), Rad6, ^His^Aos1-Uba2^His^, Ubc9^His^, ^His^Smt3 and ^His^Siz1(1-508) and human Rad6–^His^Rad18 complex were produced in *Escherichia coli* and purified as previously described (16,20–25). The yeast Rad6–^His^Rad18 complex was produced by over-expression in *S. cerevisiae* and purified as described previously (20). The Rad18 SIM* mutation (L139A, I141A and V142A) was introduced by polymerase chain reaction. ^His^Rad18(1-255) was produced in *E. coli* strain BL21-Rosetta(DE3) (Stratagene) and purified by Ni–NTA affinity, anion exchange and gel filtration chromatography. Human ^His^Uba1 was purchased from BioMol and ubiquitin was from Sigma.

The N-terminal fusion of Smt3 to yeast PCNA (^His^Smt3-PCNA) was constructed by combining Smt3 lacking the C-terminal GG motif in frame with the PCNA open reading frame in the vector pQE-POL30 (Qiagen). The protein was purified as ^His^PCNA. Human ^His^PCNA was produced in *E. coli* from plasmid pRSF-PCNA (a gift from S. Petersen-Mahrt) and purified by Ni–NTA affinity, anion exchange and gel filtration chromatography. A linear fusion of SUMO-2 to this construct was generated by inserting the sequence of SUMO-2 lacking the C-terminal GG motif between the His_6_ tag and the PCNA sequence within the same vector. The protein was purified as human ^His^PCNA. Glutathione S transferase (GST) fusion proteins used for interaction assays (GST, ^GST^Smt3, ^GST^PCNA and ^GST^Ubc9) were expressed from pGEX-4T-1 or pGEX-2TK (GE Healthcare) and purified by glutathione affinity chromatography.

### *In vitro* protein–protein interaction assays

*In vitro* interaction assays were performed in phosphate-buffered saline with 0.05% Triton X-100 by immobilizing the relevant GST-tagged protein on glutathione Sepharose for 1 h at 4°C. After washing the beads, the respective binding partner was added for a further incubation of 90 min at 4°C. The beads were then washed three times with buffer, and bound material was eluted by boiling in sodium dodecyl sulphate (SDS) sample buffer before analysing the samples by SDS-polyacrylamide gel electrophoresis (SDS-PAGE) and western blotting using an anti-His antibody (Sigma).

### *In vitro* protein modification assays

All protein names are given without specifying His_6_ tags. Unless otherwise noted, 10 µl reactions were set up in a buffer containing 40 mM HEPES, pH 7.4; 50 mM NaCl; 8 mM magnesium acetate and 1 mM adenosine triphosphate, incubated at 30°C for 1 h, terminated by addition of SDS sample buffer and denatured at 95°C for 3 min. *In vitro* sumoylation of Rad18 was analysed in reactions containing 200 nM Aos1-Uba2, 50 nM Ubc9, 20 nM Siz1(1-508), 8 µM Smt3, 2.5 nM nicked plasmid DNA and 500 nM Rad6–Rad18 complex, unless otherwise noted. Products were analysed by 8% SDS-PAGE and western blotting with polyclonal anti-Rad18 antibody. Modification of Rad6 in the same reactions was analysed by 12% SDS-PAGE and western blotting with polyclonal anti-Rad6 antibody. PCNA monoubiquitylation reactions were set up with 50 nM Uba1, 200 nM Rad6–Rad18 complex, 1 µM ubiquitin, 20 nM RFC, 3 nM nicked plasmid DNA and 50 nM PCNA trimer as described previously (20). Products were analysed by 10% SDS-PAGE and western blotting with polyclonal anti-PCNA antibody. The same conditions were applied to modification of human PCNA or SUMO-PCNA on DNA. In the absence of DNA, human PCNA was used at 500 nM. Peptide inhibition assays were performed in the presence of 30, 60 and 600 µM peptide as indicated. Peptide sequences (26) were KVDVIDLTIE (WT), KVDVADLTIE (* = mutated) and VKDVLTDEIE (sc = scrambled). Where sumoylation and ubiquitylation of yeast PCNA were analysed simultaneously, reactions additionally contained 50 nM Aos1-Uba2, 50 nM Ubc9, 8 µM Smt3 and variable amounts of Siz1(1-508) as indicated. For quantification, chemiluminescence signals were recorded on a Fuji LAS-3000 imager, and averages and standard deviations were determined from two to three independent experiments.

### Two-hybrid analysis

Two-hybrid assays for detection of *in vivo* protein–protein interactions were performed in the reporter strain PJ69-4 A as described previously (27), using the Gal4 system. Constructs for Rad18, PCNA, PCNA* and Smt3-PCNA* have been described previously (27–29), and those for Ubc9 and Smt3 were constructed analogously by insertion of the open reading frame. Protein levels were analysed in total extracts by western blotting, using antibodies against the Gal4 activation and DNA-binding domains (Clontech).

### *In vivo* analysis of yeast proteins and damage sensitivities

Total cell lysates were prepared under denaturing conditions as described previously (12,15). Rad18^9myc^ (WT or SIM*) was detected by western blotting using a monoclonal anti-myc antibody (Santa Cruz Biotechnology). For detection of PCNA ubiquitylation, cultures of appropriate *^His^POL30 (WT or K127R)* strains were treated with 0.02% methyl methanesulfonate (MMS) for 90 min where indicated, or a time course analysis was performed after addition of 0.025% MMS. Cells were lysed under denaturing conditions, ^His^PCNA was isolated by Ni–NTA affinity chromatography and conjugates were detected by anti-ubiquitin western blotting. Sensitivities towards MMS and ultraviolet (UV) (254 nm) were determined as previously described (15).

## RESULTS

### A SIM in Rad18 promotes covalent and non-covalent interactions with SUMO

**Figure 1.**
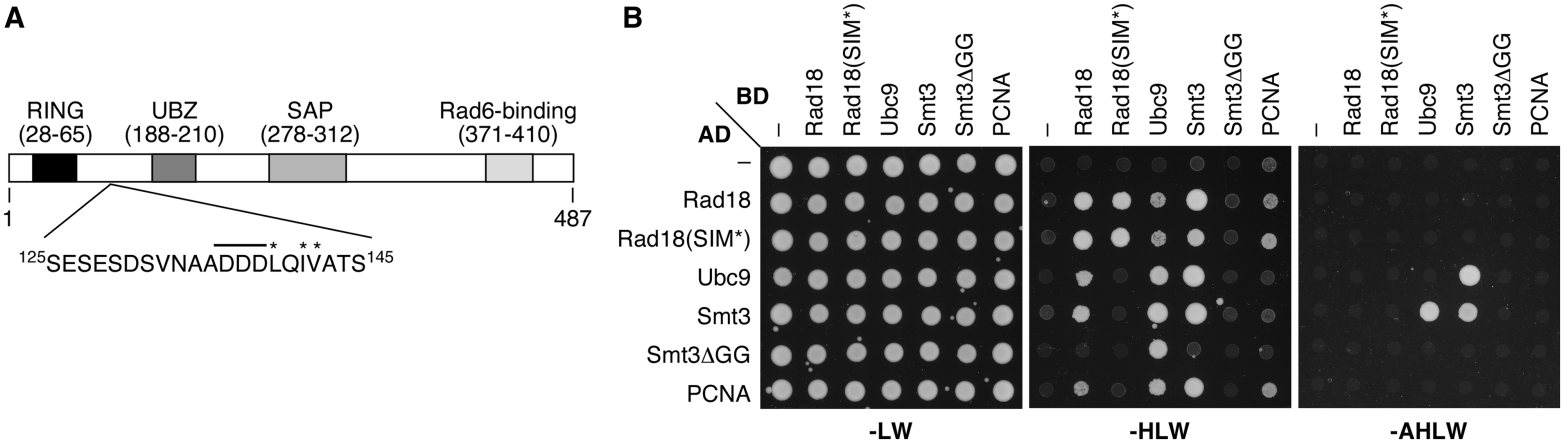
Identification of a SUMO-interacting motif in Rad18. (**A**) Domain structure of Rad18 from *S. cerevisiae*. The acidic region and the hydrophobic core of the SIM are highlighted by a bar and asterisks, respectively. (**B**) Yeast two-hybrid assay showing interactions between Rad18, Ubc9, Smt3 and PCNA. Reporter constructs, based on Gal4 activation (AD) and DNA-binding (BD) domains, confer growth on selective medium (-LW). Positive interactions were scored by growth on medium lacking histidine (-HLW) or, for stronger interactions, histidine and adenine (-AHLW). SIM*: L139A, I141A and V142A.

We noticed a sequence within budding yeast Rad18 that conforms to the consensus of a SIM, spanning amino acids 136–142 (Figure 1A). To determine whether Rad18 indeed binds to Smt3, we analysed the interaction in the two-hybrid system (Figure 1B). The assay confirmed previously reported interactions of Rad18 with itself, with Ubc9 and with PCNA (12,27). In addition, we found an association of Rad18 with full-length Smt3. Mutation of the hydrophobic core residues within the putative SIM to alanine (L139A, I141A and V142A, named SIM*) resulted in a loss of the interaction in one of the two orientations. Interactions with Ubc9 and with PCNA were affected in a similar manner. Surprisingly, truncation of the C-terminal diglycine (GG) motif in Smt3 abolished most interactions involving Smt3, suggesting that covalent rather than non-covalent interactions gave rise to these signals.

**Figure 2.**
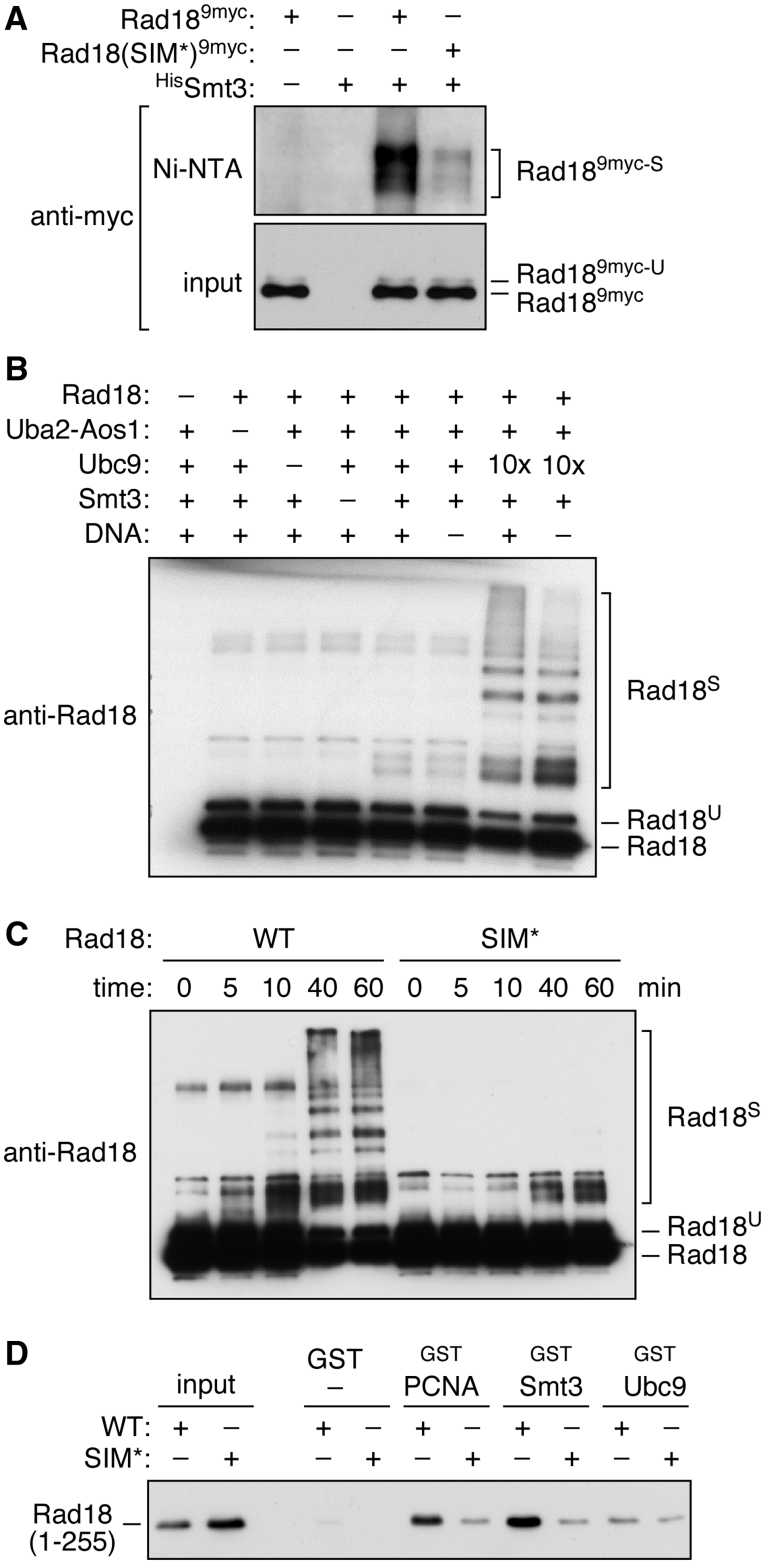
Covalent and non-covalent interactions of Rad18 with Smt3. (**A**) Rad18^9myc^ is sumoylated *in vivo* in a SIM-dependent manner. Total Smt3 conjugates were isolated by Ni–NTA pull-down from a strain expressing ^His^Smt3, and sumoylated Rad18^9myc^ (marked ‘S’) was detected in the isolated material by anti-myc western blot. Endogenous ubiquitylated Rad18 (‘U’) is also detectable. (**B**) Rad18 is sumoylated *in vitro*. Sumoylation assays were set up under standard conditions with the indicated components, and modified Rad18 was detected by western blot. The ‘10×’ indicates a 10-fold higher concentration of Ubc9. The presence of ubiquitylated Rad18 is a consequence of producing the protein in yeast. (**C**) Rad18 sumoylation is enhanced by the SIM. Time course analysis of *in vitro* sumoylation was performed with WT Rad18 and the SIM* mutant protein. (**D**) Rad18 interacts non-covalently with Smt3 in a SIM-dependent manner. ^GST^PCNA, ^GST^Smt3 or ^GST^Ubc9 was immobilized on glutathione Sepharose, and retention of ^His^Rad18(1-255), either WT or SIM*, was analysed by anti-His western blot. GST was used as a negative control.

We therefore expressed a His_7_-tagged form of Smt3 in a strain bearing a 9myc-tagged allele of *RAD18* to isolate total Smt3 conjugates under fully denaturing conditions. High-molecular-weight forms of Rad18^9myc^ were detectable in the isolated material, indicating that Rad18 is indeed covalently sumoylated *in vivo* (Figure 2A). Modification of the mutant Rad18(SIM*) protein was much reduced, although not completely abolished. Similar results were obtained *in vitro*, where addition of SUMO-specific E1 and E2 (Aos1-Uba2 and Ubc9) promoted sumoylation of purified Rad18 (Figure 2B). The reaction was independent of the presence of DNA, which is known to be required for Rad18’s ubiquitin ligase activity towards PCNA (30). Rad18’s cognate E2, Rad6, was not modified, and the SUMO ligase Siz1 moderately enhanced the reaction (Supplementary Figure S1). Importantly, the SIM* mutant was sumoylated much less efficiently (Figure 2C).

Taken together, these findings suggest that the interaction between Rad18 and Smt3 observed in the two-hybrid system was mainly caused by the sumoylation of Rad18 itself. However, as the modification was strongly dependent on the putative SIM, it is likely that this motif was responsible for directing Ubc9-bound Smt3 towards Rad18, which would imply a non-covalent association after all. We therefore investigated the interaction between Rad18 and Smt3 with recombinant proteins *in vitro*. A truncated construct that includes the putative SIM, Rad18(1-255), was efficiently retained by immobilized ^GST^Smt3 in a SIM-dependent manner (Figure 2D). This indicates that the motif indeed mediates non-covalent interaction of Rad18 with Smt3. The weak interaction observed with the SIM* mutant might be because of either a residual affinity of the mutated SIM or a second unidentified binding site in the construct. As observed in the two-hybrid system, the interaction between Rad18 and PCNA was also affected by mutation of the SIM, whereas recombinant Ubc9 bound to Rad18 weakly, but in a SIM-independent manner. Overall, our data show that Rad18 interacts non-covalently with Smt3 via a canonical SIM, which also promotes the covalent sumoylation of Rad18 *in vivo* and *in vitro*.

### The SIM directs Rad18’s ubiquitin ligase activity towards sumoylated PCNA

**Figure 3.**
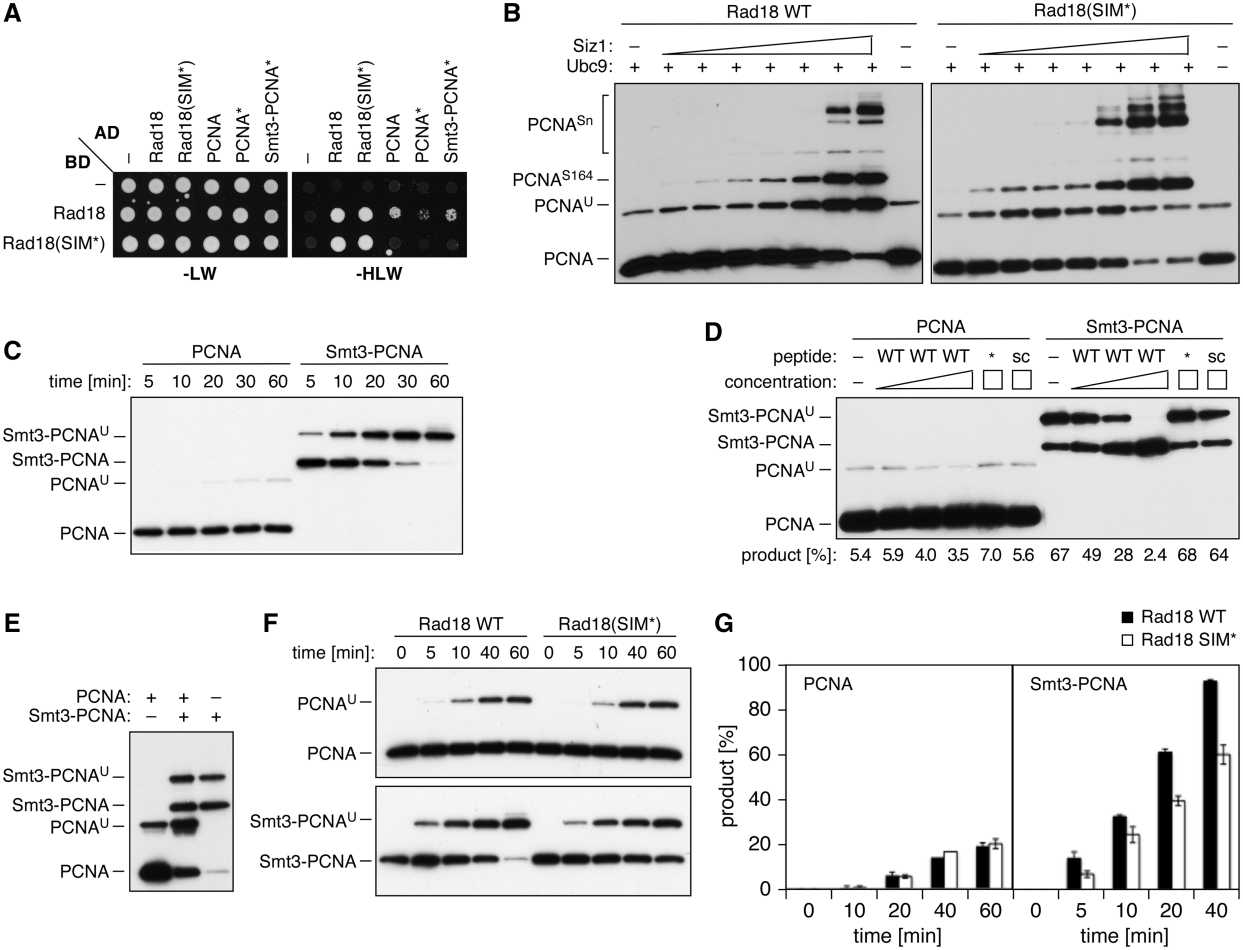
Interactions and activities of Rad18 towards sumoylated PCNA. (**A**) Fusion of Smt3 to PCNA enhances interaction with Rad18. Two-hybrid analysis was performed as in Figure 1B. The mutant PCNA(K127/164R) is labelled PCNA*. Expression levels of the constructs are shown in Figure S2. (**B**) Ongoing sumoylation enhances the ubiquitin ligase activity of Rad18, but not Rad18(SIM*), towards PCNA. *In vitro* modification reactions were set up with all factors required for PCNA ubiquitylation, as well as Aos1-Uba2 and Smt3. Siz1 concentrations were varied from 0.8 to 50 nM. Products were detected by anti-PCNA western blot. (**C–F**) Standard *in vitro* ubiquitylation reactions, analysed as earlier in text. (C) Time course analysis of *in vitro* ubiquitylation of PCNA and Smt3-PCNA. (D) Reversal of the stimulatory effect of Smt3 by a canonical SIM peptide (WT), but not a mutated (*) or scrambled (sc) version. Peptide concentrations ranged from 30 to 600 µM, and the amount of product formed is indicated below the blot. (E) Presence of Smt3-PCNA enhances ubiquitylation of native PCNA. (F) The enhanced activity of Rad18 towards Smt3-PCNA relies on an intact SIM. Time course analysis was performed with PCNA (upper panel) and Smt3-PCNA (lower panel), using Rad18 WT or the SIM* mutant protein. (**G**) Quantification of the blots shown in panel F.

The notion that a significant portion of PCNA is sumoylated during S phase (24) raised the question of whether the SIM would mediate a preferential interaction of Rad18 with the sumoylated form of PCNA. We addressed this in the two-hybrid system. When compared with wild-type PCNA, a mutant that cannot be modified by ubiquitin or SUMO, PCNA(K127/164R), designated as PCNA*, gave a reduced interaction signal with Rad18, suggesting that sumoylation of PCNA within the two-hybrid construct might be important for the association (Figure 3A, Supplementary Figure S2). Consistent with this notion, covalent fusion of Smt3 to the N-terminus of PCNA* restored the interaction. This result prompted us to compare the catalytic activity of Rad18 as a ubiquitin ligase towards PCNA in the presence or absence of Smt3 *in vitro*. We first followed the extent of PCNA ubiquitylation *in vitro* under conditions where the clamp would also be sumoylated. In the presence of all the required ubiquitylation components and the SUMO-specific E1 and E2 enzymes, increasing amounts of the SUMO E3, Siz1, resulted in an enhancement of PCNA ubiquitylation, roughly parallel to the amount of sumoylated PCNA that was produced (Figure 3B). Use of the Rad18(SIM*) mutant abolished the enhancement of PCNA ubiquitylation by Siz1 without decreasing the efficiency of sumoylation. To verify that this effect was attributable to the conjugation of SUMO to PCNA and not to any other reaction component, such as Rad18 itself, we examined *in vitro* ubiquitylation of a linear fusion of Smt3 to the N-terminus of PCNA in the absence of any sumoylation enzymes. Compared with native PCNA, the Smt3 fusion was ubiquitylated with strongly enhanced efficiency, as evident from time course experiments (Figure 3C), as well as titrations of Rad18 (Supplementary Figure S3A). The stimulating effect of the Smt3 moiety was reversed by the addition of an excess of a canonical SIM peptide derived from the PIAS2 protein (26), but not a mutated or scrambled version, indicating that it is indeed caused by a genuine SIM–SUMO interaction (Figure 3D). When a mixture of native PCNA and Smt3-PCNA was used as a substrate, ubiquitylation was stimulated not only on the fusion protein but also on native PCNA (Figure 3E). Considering that the two species can form mixed trimers, this result suggests that the position of Smt3 within the PCNA ring is not important for its stimulatory effect.

A number of controls demonstrated that the Smt3-stimulated activity of Rad18 closely reflects its natural behaviour towards PCNA: Rad6 in the absence of Rad18 was unable to modify PCNA or Smt3-PCNA (Supplementary Figure S3B), the reaction required RFC-dependent loading of the substrate onto DNA (Supplementary Figure S3C), and ubiquitylation required the physiological site, K164 (Supplementary Figure S3D). Fusion of ubiquitin instead of Smt3 to the N-terminus of PCNA afforded comparatively minor stimulation (Supplementary Figure S3E). Importantly, although WT and SIM* proteins exhibited similar activities towards native PCNA, fusion of Smt3 to PCNA resulted in a much larger enhancement of the reaction for WT Rad18 compared with the mutant (Figure 3F and G and Supplementary S3F). Again, a residual affinity for Smt3 (Figures 1B and 2D) was likely responsible for the moderate effect of the Smt3 moiety on the activity of Rad18(SIM*). Taken together, these results imply that Rad18’s SIM is capable of promoting a productive interaction with an Smt3 moiety on the PCNA trimer, resulting in a strongly enhanced ubiquitin ligase activity.

### The SIM–SUMO interaction contributes to Rad18 function *in vivo*

**Figure 4.**
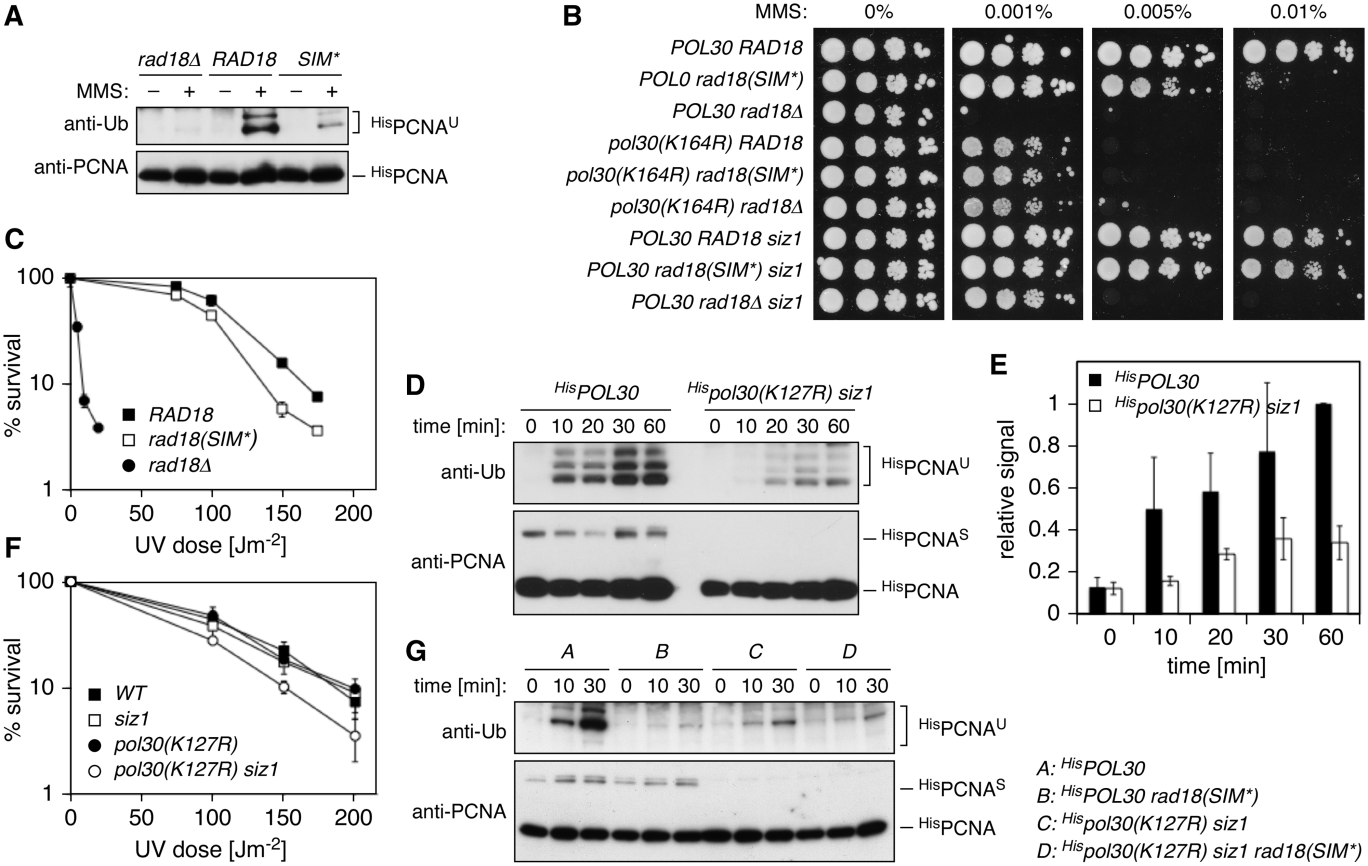
Relevance of the SIM–SUMO interaction for Rad18 function *in vivo*. (**A**) Damage-induced PCNA ubiquitylation is reduced in the SIM* mutant. ^His^PCNA was isolated by Ni–NTA pull-down under denaturing conditions from extracts of MMS-treated cells, and ubiquitin conjugates were detected by anti-ubiquitin western blot. (**B**) Mutation of the SIM confers enhanced sensitivity to MMS in a manner dependent on K164 of PCNA, but the phenotype is suppressed by deletion of *SIZ1*. Deletion mutants of *rad18* were complemented with *RAD18* (WT or SIM*) for spot assays. An empty vector served as control. (**C**) Mutation of the SIM confers enhanced UV sensitivity. (**D**) Damage-induced PCNA ubiquitylation is reduced in a mutant deficient in PCNA sumoylation, *pol30(K127R) siz1*. A modification time course was analysed as in panel A. (**E**) Quantification of the blots shown in panel D, relative to the *WT* signal at 60 min. (**F**) The *pol30(K127R) siz1* mutant displays enhanced UV sensitivity. (**G**) The PCNA ubiquitylation defects of rad18(SIM*) and *pol30(K127R) siz1* show an epistatic relationship. Modifications were analysed as in D.

Consistent with a relevance of the SIM for Rad18 function *in vivo*, damage-induced PCNA ubiquitylation was reduced in the mutant (Figure 4A), and the cells exhibited moderate sensitivity towards the alkylating agent MMS and UV radiation (Figure 4B and C). Mutation of K164 of PCNA abolished this effect, confirming that the damage sensitivity is specific to a defect in PCNA ubiquitylation (Figure 4B). Deletion of *SIZ1* suppressed the phenotype of rad18(SIM*), as expected from the notion that a failure to recruit Srs2 allows damage processing by homologous recombination. The partial loss of function was not likely caused by a destabilization of the protein, as equal levels of wild-type and mutant Rad18^9myc^ were detectable in total cell extracts (Figure 2A). Yet, despite a WT-like activity towards native PCNA *in vitro* (Figure 3F), we had observed a reduced interaction of the mutated protein with PCNA (Figures 1B and 2D). To rule out that this reduction in PCNA binding was causing the phenotype of the mutant *in vivo*, we examined a strain bearing *WT RAD18*, but devoid of PCNA sumoylation, *pol30(K127R) siz1*. In this strain, PCNA ubiquitylation was also reduced >3-fold (Figure 4D and E), and cells again exhibited UV sensitivity (Figure 4F). Combination of these mutations with rad18(SIM*) did not lead to a loss of PCNA ubiquitylation beyond the level of the rad18(SIM*) single mutant, demonstrating an epistatic relationship between the two defects (Figure 4G). Hence, a functional SIM–SUMO interaction contributes to full activity of Rad18 towards PCNA, and its loss gives rise to a phenotype comparable with that of mutants in other components of the pathway, such as *rev3* or *rad30* (15).

### Preference for sumoylated PCNA is not a conserved feature of Rad18

**Figure 5.**
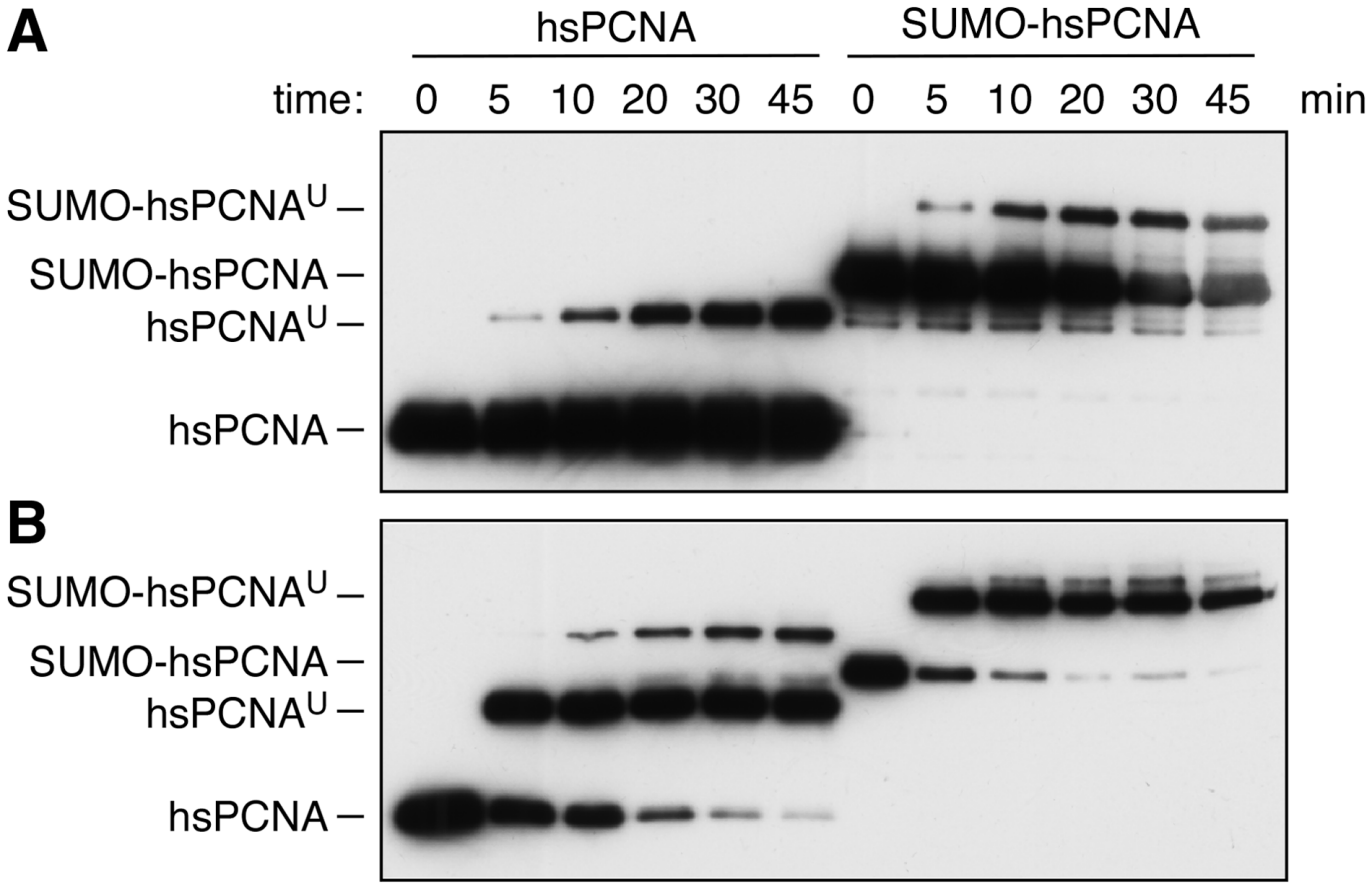
Human Rad18 is unaffected by fusion of SUMO to PCNA. *In vitro* ubiquitylation reactions were set up with human Rad6–Rad18 complex and human PCNA or a linear fusion with SUMO-2. Products were detected by western blot with an antibody against human PCNA. (**A**) Reactions set up without DNA and RFC. (**B**) Reactions set up in the presence of DNA and RFC.

We asked whether the stimulation of Rad18 activity by the presence of a SUMO moiety on PCNA was a conserved phenomenon. SUMO-2 was therefore fused to the N-terminus of human PCNA, and the construct was used as a substrate for the human Rad6–Rad18 complex, either free in solution (Figure 5A) or loaded onto DNA (Figure 5B). As in the yeast system, RFC and DNA strongly stimulated the overall efficiency of the reaction. However, the presence of SUMO on PCNA did not enhance Rad18’s activity, consistent with the lack of an obvious SIM in the human Rad18 sequence. Although sumoylated PCNA has recently been observed in human cells, its abundance is low, and its regulation may well differ from the situation in yeast (31). Hence, the observed phenomenon does not seem to be a universal property of Rad18. Rather, it appears that the SIM in Rad18 from *S. cerevisiae* is a feature that enables the ligase to specifically react to this abundant modification of chromatin-bound PCNA and facilitates the transition to the damage-associated state.

## DISCUSSION

### Implications of SUMO binding for Rad18 function in DNA damage bypass

Based on these observations, we propose that a DNA-bound PCNA trimer bearing at least one SUMO moiety is the physiological substrate of Rad18 in *S. cerevisiae*. As we previously showed, a significant portion of the total cellular PCNA pool is sumoylated during DNA replication, and—similar to ubiquitylation—its residence on DNA is a prerequisite for sumoylation (24). Hence, our model implies a direct transition from sumoylation to ubiquitylation on replication problems or DNA damage, without an intervening desumoylation step. Isolation of a species bearing SUMO on K127 and ubiquitin on K164 within the same subunit indicates that the two modifiers can coexist on a single clamp *in vivo* (16).

This model stipulates that neither sumoylation nor ubiquitylation affects all three subunit of the PCNA trimer in concert. Although initial observations had suggested that ubiquitin can be conjugated to all three subunits of the clamp in damage-treated human cells (14), indirect evidence from yeast and mouse PCNA lends support to our model: we have shown that co-expression of a linear ubiquitin–PCNA fusion and a native non-modifiable PCNA allele supports translesion synthesis, despite the low abundance of fully ‘ubiquitylated’ trimers expected from a stochastic arrangement (28). Along the same lines, normal activation of the damage-tolerant DNA polymerase η in heterozygous PCNA^K164R^ murine B cells suggests that modification of all three subunits within a trimer is not required for ubiquitin-dependent translesion synthesis (32).

Thus, Rad18 activity towards PCNA appears to be controlled on two levels: on the one hand, we previously showed that the replication protein A complex recruits the E3 to sites where replication problems have caused the accumulation of single-stranded DNA (19); on the other hand, the SIM–SUMO interaction described here directly enhances the recognition of PCNA as a relevant substrate. In this way, the properties of budding yeast Rad18 not only ensure an ordered transition from sumoylated to ubiquitylated PCNA but they may also limit inappropriate PCNA modification under conditions where the clamp is not engaged in replication.

### Relevance of SIM-dependent Rad18 sumoylation

The SIM not only stimulates ubiquitin ligase activity of Rad18 towards sumoylated PCNA but also enhances the sumoylation of Rad18 itself. The reaction is reminiscent of a phenomenon called coupled monoubiquitylation, which is based on the interaction of a ubiquitylation target with a ubiquitin-charged E2 or a ubiquitylated E3, mediated by a ubiquitin-binding domain within the substrate (33,34). Whether Rad18’s SIM recognizes the thioester-bound modifier or a SUMO moiety attached to a lysine on Ubc9 (35) is unknown, but the reaction does not require a SUMO ligase. In the two-hybrid system, mutation of the Rad18 SIM results in a partial loss of interaction with Ubc9 (Figure 1B), and in pull-down assays the SIM-independent affinity of Rad18 for unmodified Ubc9 is low (Figure 2D), indicating that the contact between Rad18 and Ubc9 is largely mediated by SUMO. Given that SUMO conjugates of Rad18 are much less abundant *in vivo* than the ubiquitylated forms of the E3 (Figure 2A), sumoylation might be an inevitable consequence of SUMO binding and of little physiological relevance for Rad18 function. This, however, will need to be examined in a future study.

### Substrate recognition by Rad18 and other STUbLs

Based on the properties of its SIM, Rad18 exhibits the hallmarks of a STUbL. Although our data show that interactions with both SUMO and PCNA contribute to the efficient recognition of sumoylated PCNA by Rad18, the situation is not always that clear for other STUbLs. Most notably, few physiological substrates have so far been identified. Prominent examples are the sumoylated forms of human promyelocytic leukaemia protein PML or the PML–RARα fusion, which are ubiquitylated by RNF4; however, the E3 predominantly recognizes these substrates via their poly-SUMO chains (7). *In vitro*, STUbLs often act relatively unspecifically ubiquitylating artificial test substrates such as GST-SUMO, sumoylated Siz2 or free poly-SUMO chains (6,36). There are a few notable exceptions to this rule: budding yeast Slx5–Slx8 complex mediates the ubiquitylation and subsequent degradation of the transcription factor Matα2 (37), and the *Drosophila* STUbL, Degringolade, ubiquitylates the transcriptional repressor Hairy (38). Intriguingly, sumoylation was found to be dispensable for ubiquitylation in both cases, suggesting that STUbLs can also function in a SIM-independent manner. In terms of substrate properties, the transcription factor Mot1 from *S. cerevisiae* may resemble Rad18 most closely, as the Slx5–Slx8 complex recognizes this substrate through SUMO, as well as epitopes on the protein itself (39). However, ubiquitylation apparently correlated with misfolding in this case, suggesting a possible role of the STUbL in general protein quality control rather than the targeting of specific substrates. Clearly, much remains to be learned about the mechanisms by which STUbLs select their targets. Our identification of budding yeast Rad18 as a substrate-specific member of this enzyme family has given insight into a new aspect of STUbL function by showing how the enzyme can coordinate an ordered progression from one modification to another.

## SUPPLEMENTARY DATA

Supplementary Data are available at NAR Online: Supplementary Figures 1–3.

## FUNDING

Funding for open access charge: Cancer Research UK [A7123].

*Conflict of interest statement*. None declared.

## Supplementary Material

Supplementary Data
