# Supplementary material for: A SUMO-interacting motif activates budding yeast ubiquitin ligase Rad18 towards SUMO-modified PCNA
Source: Nucleic Acids Res. 2012 Oct 2;40(22):11380–8. doi: 10.1093/nar/gks892 (PMC3526273; doi:10.1093/nar/gks892)
Supplement: Supplementary Data [file supp_40_22_11380__index.html]

A SUMO-interacting motif activates budding yeast ubiquitin ligase Rad18 towards SUMO-modified PCNA — A SUMO-interacting motif activates budding yeast ubiquitin ligase Rad18 towards SUMO-modified PCNA — Supplementary Data 

# A SUMO-interacting motif activates budding yeast ubiquitin ligase Rad18 towards SUMO-modified PCNA

## Supplementary Data

files

**Files in this Data Supplement:**

- Supplementary Data - pdf file
