# Supplementary material for: A SUMO-interacting motif activates budding yeast ubiquitin ligase Rad18 towards SUMO-modified PCNA
Source: Nucleic Acids Res. 2012 Oct 2;40(22):11380–8. doi: 10.1093/nar/gks892 (PMC3526273; doi:10.1093/nar/gks892)
Supplement: Supplementary Data [file supp_gks892_nar-01181-d-2012-File007.pdf]

Supplementary Information

A SUMO-interacting motif activates budding yeast ubiquitin ligase Rad18 towards SUMO-modified PCNA

Joanne L. Parker and Helle D. Ulrich

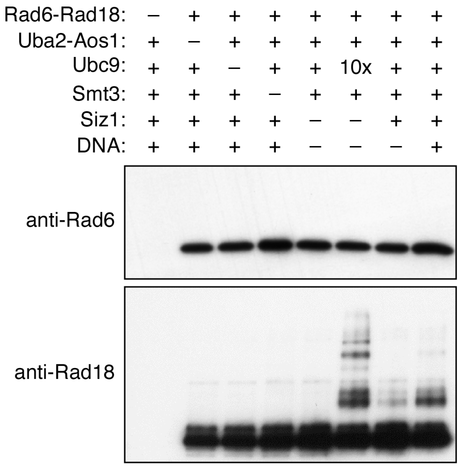

Figure S1. Sumoylation of Rad18 in vitro is moderately enhanced by Siz1, but Rad6 is not modified in the reaction. Sumoylation assays were set up under standard conditions with the indicated components. Rad6 (upper panel) and Rad18 (lower panel) were detected by Western blot. “10x” indicates a 10-fold higher concentration of Ubc9.

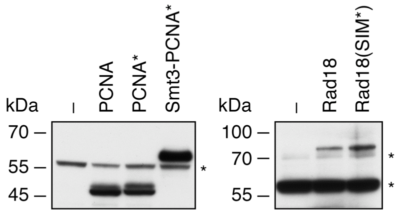

Figure S2. Protein levels of the two-hybrid constructs used for analysis of the Rad18-Smt3-PCNA interactions. Total cell extracts were analysed by Western blot with antibodies against the Gal4 activation and DNA-binding domains to reveal the fusion proteins. Asterisks indicate cross-reacting bands.

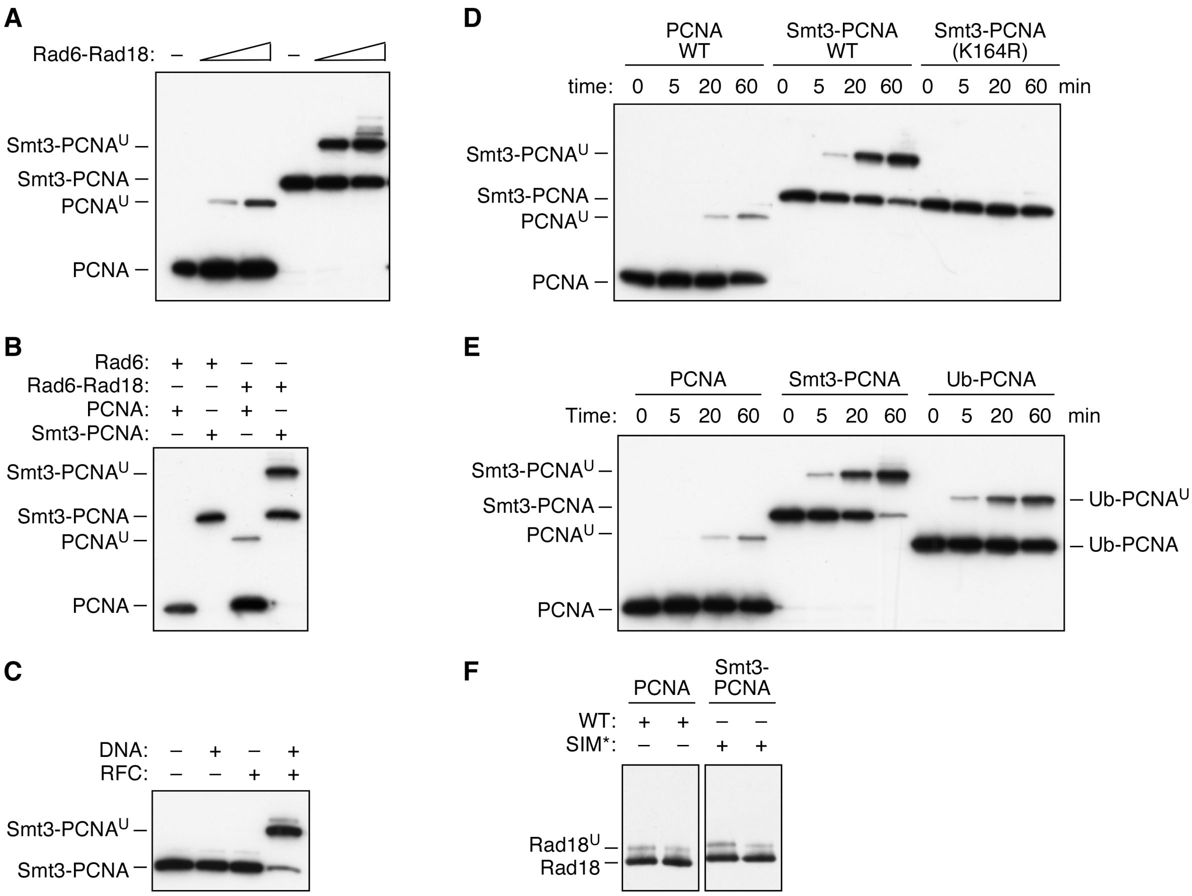

Figure S3. Effects of Smt3 on ubiquitylation of PCNA by Rad18 in vitro. Standard in vitro ubiquitylation reactions were set up as indicated and analysed by anti-PCNA Western blot. (**A**) Covalent fusion of Smt3 to PCNA enhances ubiquitylation by Rad18. Rad6-Rad18 were used at 150 nM and 300 nM. (**B**) Rad6 alone does not promote ubiquitylation of PCNA or Smt3-PCNA. Reactions were set up with equimolar amounts of either Rad6 or Rad6-Rad18. (**C**) RFC-dependent clamp loading onto DNA is required for ubiquitylation of Smt3-PCNA. (**D**) Ubiquitylation of Smt3-PCNA occurs at K164 of PCNA. Time course analysis was performed with the indicated proteins. (**E**) Linear fusion of ubiquitin to the N-terminus of PCNA provides minor stimulation of ubiquitylation by Rad6-Rad18, compared to fusion of Smt3. Note that the presence of a ubiquitin-binding UBZ domain in Rad18 might be responsible for this effect. (**F**) Anti-Rad18 Western blots of the reactions shown in Figure 4E at time zero, indicating equal amounts of Rad18 proteins.
